# Compositional Analysis of Probabilistic Timed Graph Transformation Systems

**DOI:** 10.1007/978-3-030-71500-7_10

**Published:** 2021-02-24

**Authors:** Maria Maximova, Sven Schneider, Holger Giese

**Affiliations:** 8 Universidad Autónoma de Madrid, Madrid, Spain; 9 University of Twente, Enschede, The Netherlands; Hasso Plattner Institute, University of Potsdam, Potsdam, Germany

**Keywords:** cyber-physical systems, graph transformation systems, qualitative analysis, quantitative analysis, probabilistic timed systems, compositional analysis, model checking

## Abstract

The analysis of behavioral models is of high importance for cyber-physical systems, as the systems often encompass complex behavior based on e.g. concurrent components with mutual exclusion or probabilistic failures on demand. The rule-based formalism of probabilistic timed graph transformation systems is a suitable choice when the models representing states of the system can be understood as graphs and timed and probabilistic behavior is important. However, model checking PTGTSs is limited to systems with rather small state spaces.

We present an approach for the analysis of large-scale systems modeled as probabilistic timed graph transformation systems by systematically decomposing their state spaces into manageable fragments. To obtain qualitative and quantitative analysis results for a large-scale system, we verify that results obtained for its fragments serve as overapproximations for the corresponding results of the large-scale system. Hence, our approach allows for the detection of violations of qualitative and quantitative safety properties for the large-scale system under analysis. We consider a running example in which we model shuttles driving on tracks of a large-scale topology and for which we verify that shuttles never collide and are unlikely to execute emergency brakes. In our evaluation, we apply an implementation of our approach to the running example.

## 1 Introduction

Real-time cyber-physical systems often emit a complex behavior based on e.g. concurrent components with mutual exclusion or probabilistic failures on demand. Consequently, modeling formalisms for capturing such systems must suitably support the modeling of their complex behaviors. In such a model driven approach, the analysis of behavioral models w.r.t. a provided specification is vital to ensure overall soundness of the resulting system.

* Funded by the Deutsche Forschungsgemeinschaft (DFG, German Research Foundation) - 241885098, 148420506.

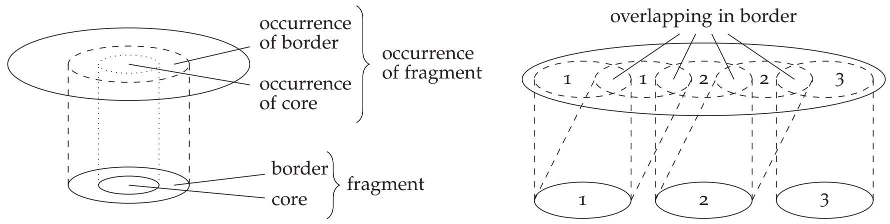

Fig. 1: Occurrence of single FT with border and core in LST (left) and five occurrences of three FTs in LST overlapping in their borders (right).

The rule-based transformation of graphs is a suitable choice when the models representing states of the system can be understood as graphs. In particular, the formalism of probabilistic timed graph transformation systems (PTGTSs) extends the standard rule-based transformation of graphs such that timed and probabilistic behavior is covered by supporting (a) non-deterministic choice among steps, (b) probabilistic choice among step results, and (c) steps representing the passage of time.

A model checking approach for PTGTSs w.r.t. probabilistic metric temporal properties was introduced in [19]. However, also this model checking approach is limited to systems with rather small state spaces due to the state space explosion problem. As a workaround, a selected set of small examples may be considered hopefully capturing all system-specific challenges to establish trust that the model exhibits the required safe behavior and that unwanted behavior is sufficiently unlikely. However, it cannot be excluded that the considered small examples do not reveal all the threatening behavior.

We present a decomposition-based approach for the analysis of large-scale systems modeled as PTGTSs to rule out violations of qualitative and quantitative safety properties.

As a first step, we capture the underlying static large-scale topology (short LST) of a large-scale system as a subgraph that is not changed by graph transformation, describe how a fragment topology (short FT) can be embedded into such an LST (see the left part of Figure 1), and specify how multiple such embeddings of FTs can overlap in their borders (see the right part of Figure 1).

As a second step, based on the decomposition described by such embeddings, we construct for each FT an adapted PTGTS. Such an adapted PTGTS is then ensured to (a) exhibit the same behavior on the non-overlapped part of the FT (named *core*) and to (b) simulate all possible behaviors that can happen for any occurrence of the FT in an LST. To obtain the mentioned simulation, we include modifications of the rules of the original PTGTS operating on the border of an FT into the adapted PTGTS. With this direct relationship between behaviors on the FTs and the LST, we obtain that the likelihood of an unwanted or forbidden graph pattern in one of the adapated PTGTS is an upper bound for its likelihood in its embedding in the large-scale PTGTS.

As a last step, exploiting our decomposition to counter the state space explosion problem, we apply the model checking approach from [19] to the PT-

GTSs constructed for the FTs employing its reduction to probabilistic timed automata (PTA) instead of applying the model checking approach directly to the PTGTS modeling the large-scale system.

To illustrate our approach, we consider a running example in which we model shuttles driving on tracks of an LST and for which we verify that shuttles never collide and are unlikely to execute emergency brakes. In our evaluation, we apply an implementation of our approach to the running example.

The idea to decompose a system into subsystems or to compose it from subsystems for the analysis has been studied intensively [25] but our suggested compositional approach has distinguishing characteristics. Firstly, the vast majority of approaches (like process algebras or similar models) assume that the modeling formalism supports the composition/decomposition as a first class concept such that compositional analysis techniques are directly applicable as the subsystem models cover all possible behaviors in all contexts. In contrast, we do not rely on a built-in decomposition operator but rather allow for a flexible derivation of an LST decomposition in terms of FTs, overlappings, and a suitable overapproximation on the border, which are not predefined by the modeling formalism.

Secondly, several approaches rely on a protocol-like specification of how the decomposed subsystems interact, while in our approach the overapproximation is derived systematically from the PTGTS model that does not necessarily provide such a protocol-like specification already. The compositional analysis approach for graph transformation systems (GTSs) from [24, 11] defines explicit interfaces, which are used to consider whether the behavior of two independent graphs glued via these interfaces (requiring that local transitions are compatible) cover jointly all global transitions. Moreover, in further approaches, protocols for the roles of collaborations and ports of components have been assumed. For example, in [14], the idea to overapproximate the environment and border is explored for timed automata with explicit models of the roles in form of protocol automata. This idea has been combined with dynamic collaborations in [12, 13] captured by timed GTSs (TGTSs) and their analysis via inductive invariant checking [3, 4]. Later on, this approach has been extended to role, component, and collaboration behavior, which is captured by TGTSs and hybrid GTSs in [5] and [2], respectively. However, as opposed to the presented approach, in all these cases an explicit concept of interface is assumed to separate parts that are analyzed in isolation.

This paper is structured as follows. In section 2, we introduce our running example from the domain of cyber-physical systems. In section 3, we recapitulate the necessary preliminaries related to PTA and PTGTSs also presenting the modeling of our running example. In section 4, we discuss the decomposition of static substructures of large-scale systems. In section 5, we present our decomposition-based approach allowing to split the model checking problem into more manageable parts. In section 6, we present an evaluation of the conceptual results for our running example. Finally, in section 7, we close the paper with a conclusion and an outlook on planned future work.

## 2 Running Example

We now informally introduce a scenario (based on the RailCab project [23]) of autonomous shuttles driving on an LST, which serves as a running example in the remainder of this paper. Based on this introduction, we will discuss how we model this shuttle scenario as a PTGTS in the next section.

In the considered shuttle scenario, a track topology containing a large number of tracks of approximately equal length is given. Tracks are connected to the adjacent tracks via directed connections building in this manner track sequences. Two track sequences can be joined together (i.e., can end up in a common track with two predecessors) leading to a *join* fragment topology (see FT8 in Figure 4a) or can split up from a common track (i.e., a common track has then two successor tracks) leading to a *fork* fragment topology (see FT7 in Figure 4a). Moreover, depots may have a directed connection to a track allowing shuttles to enter or exit the track topology. Shuttles, which are always located on a single track, may be in mode *DRIVE*, *STOP*, or *BRAKE*. Being in mode *DRIVE*, shuttles drive to the next track (respecting the direction of the connection between the tracks) with a certain velocity, which may be slow ($[3, 4]$ time units per track) or fast ($[2, 3]$ time units per track). Regularly, shuttles change into mode *STOP*, which allows them to avoid coming too close to other shuttles. Moreover, shuttles should slow down before entering a track with a construction site on it. However, shuttles noticing the construction site too late have to execute an emergency brake thereby changing into the mode *BRAKE*. To reduce the likelihood of such emergency brakes, yellow traffic lights are installed a few tracks ahead of such construction sites to indicate to shuttles that they should slow down. After construction sites, green traffic lights may be installed permitting shuttles to increase their velocity. However, we also consider failures on demand where a traffic light that is passed by a shuttle is not recognized or, for some other reason, not appropriately taken into account by the shuttle. We assume a failure probability of $10^{-6}$ for this case assuming that the failure does not only depend on the visual observation by the train driver but also depends on a failure of the backup system.

In our running example, *static* elements are the tracks, depots, installed traffic lights, and construction sites as well as connections between these elements. The PTGTS modeling the behavior of the described scenario never changes this underlying LST. Complementary, *dynamic* elements are shuttles, their attributes, their connections to tracks of the LST as well as the attributes of traffic lights. Note that we use later a grammar to generate admissible LSTs.

For the considered shuttle scenario, we are interested in various properties. Firstly, we need to verify that the behavior of the system never gets temporally stuck in a state where no steps (discrete steps of e.g. driving shuttles or timed steps) are enabled. Secondly, we need to verify whether the rules have been constructed in a way ensuring the absence of collisions between shuttles (i.e., two shuttles should not be on a common track). Thirdly, emergency brakes should be improbable at a local level for a single shuttle but also at the global level for the entire LST and its possible numerous number of shuttles.

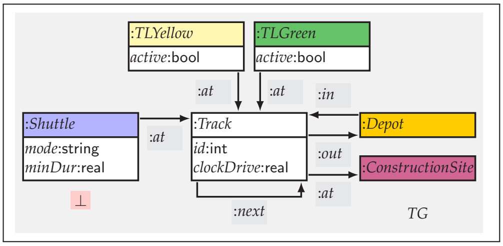

(a) Type graph

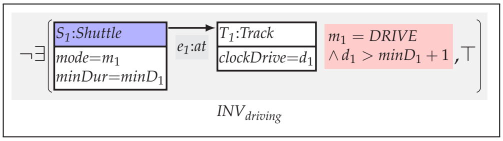

(b) Invariant

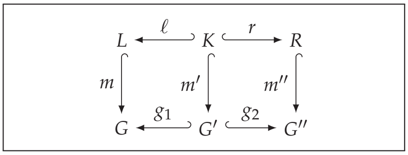

(c) DPO diagram

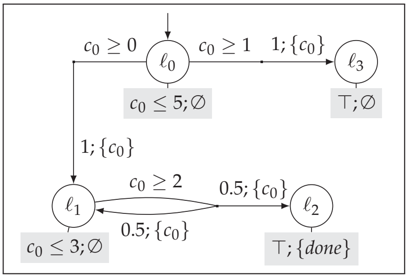

(d) Example of a PTA

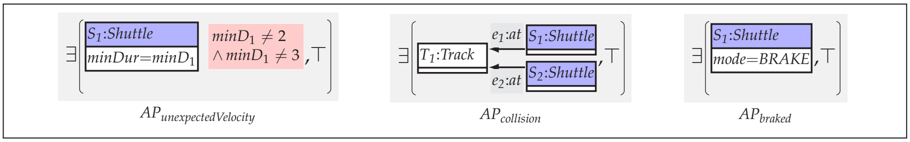

(e) Atomic propositions

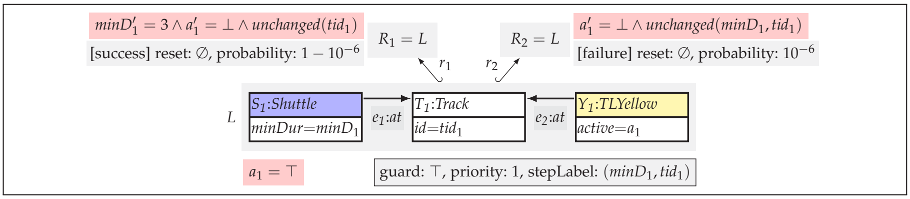

(f) The rule *SetSlow*: a shuttle may *successfully* decrease its velocity by setting its time per track to $[3, 4]$ (where only the lower end of the interval is stored in the graph) with probability $1 - 10^{-6}$ or may *fail* to decrease its velocity with probability $10^{-6}$. Setting the *active* attribute to $\perp$ ensures that the rule cannot be applied twice.

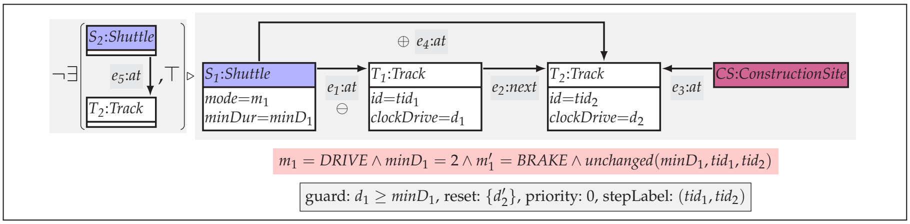

(g) The rule *ConstructionSiteBrake*: a shuttle with high velocity ($[2, 3]$ time units per track where only the lower end of the interval is stored in the graph) needs to execute an emergency brake to ensure that the track with a construction site on it is not entered with a too high velocity.

Fig. 2: Details for our running example, DPO diagram, and PTA example.

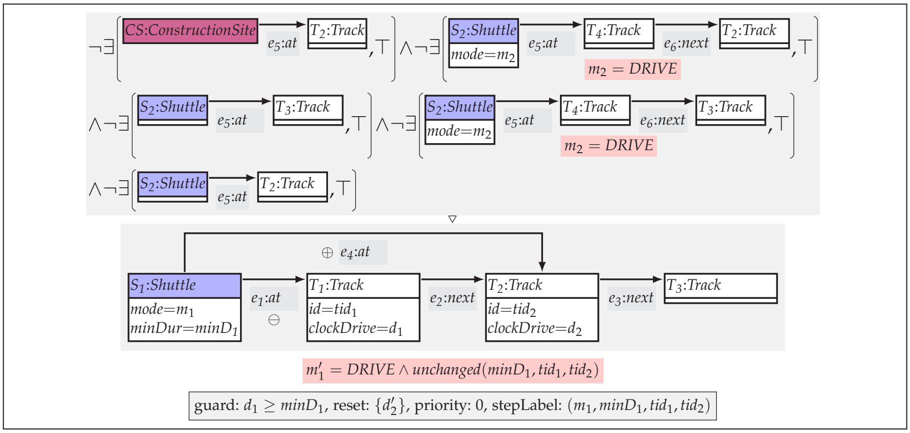

(a) The rule *Drive*: a shuttle may drive to the next track where the application condition is used to rule out situations that on the next track is a construction site or that the considered shuttle comes too close to another shuttle.

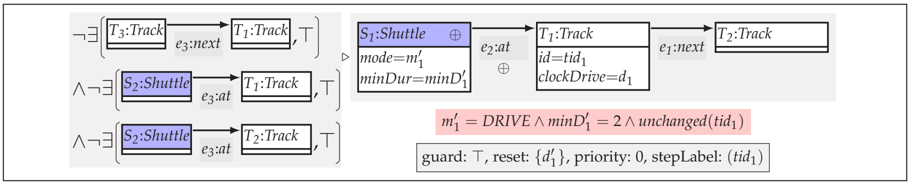

(b) The rule *DriveEnterFast*: adaptation of the rule *Drive* for the case that a new shuttle enters the current fragment topology with a high velocity (the similar rule for a shuttle with a low velocity has been omitted here for brevity) from a context track belonging to another fragment topology.

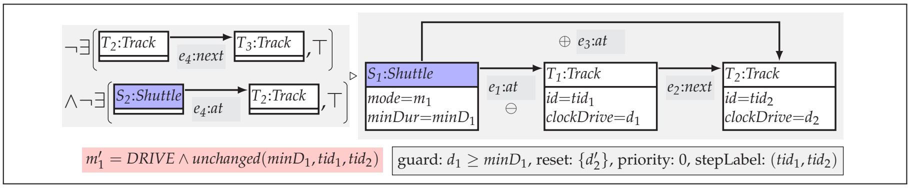

(c) The rule *DriveExit1*: adaptation of the rule *Drive* for the case that a shuttle drives onto the last track of the current fragment topology.

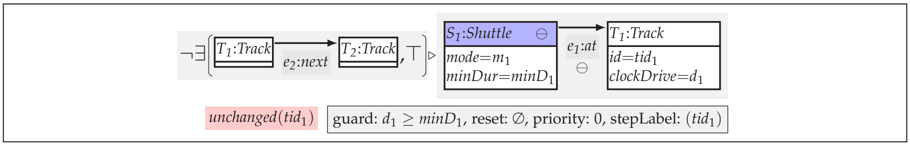

(d) The rule *DriveExit2*: adaptation of the rule *Drive* for the case that a shuttle exits the current fragment topology towards a track belonging to another fragment topology.

Fig. 3: The rule *Drive* and the three adapted rules *DriveEnterFast*, *DriveExit1*, and *DriveExit2* for fragment topologies where parts of the application condition of the rule *Drive* are omitted due to the overlay specification of the running example.

## 3 Preliminaries

We now briefly introduce the subsequently required details for graph transformation systems (GTSs) [10], probabilistic timed automata (PTA) [17], and probabilistic timed graph transformation systems (PTGTSs) [18, 19] in our notation. Along this presentation, we also discuss the modeling details for our running example from the previous section.

We employ type graphs (cf. [10]) such as the type graph $TG$ from Figure 2a for our running example. A type graph describes the set of all admissible (typed attributed) graphs by mentioning the allowed types of nodes, edges, and attributes. We assume typed attributed graphs in which attributes are specified using a many sorted first-order attribute logic as proposed in [21] (the attribute constraint $\bot$ (false) in $TG$ means that the type graph does not restrict attribute values). This approach to attribution has been used to capture constraints on attributes in graph conditions in [27] and to describe attribute modifications in [22, 28].

Graph transformation is then performed by applying a graph transformation rule (short rule) $\rho = (\ell : K \hookrightarrow L, r : K \hookrightarrow R)$ consisting of two monomorphisms (i.e., all components of the morphisms are injective). The rule specifies that the graph elements in $L - \ell(K)$ are to be deleted, the graph elements in $K$ are to be preserved, and the graph elements in $R - r(K)$ are to be added during graph transformation. Such a rule is applied to a graph $G$ for a given match $m : L \hookrightarrow G$ resulting in a graph $G''$ by constructing the double pushout (DPO) diagram (see Figure 2c) where the first and the second pushout squares describe the removal and the addition of graph elements specified in the rule, respectively. Moreover, a rule may additionally contain an application condition $\phi$ (denoted by $\rho = (\ell, r, \phi)$) to rule out certain matches specifying e.g. graph elements that may not be connected to graph elements matched by $m$. For further details on the graph transformation approach, we refer to [10].

PTA [17] combine the use of clocks to capture real-time phenomena and probabilism to approximate/describe the likelihood of outcomes of certain steps. A PTA such as the one in Figure 2d consists of (a) a set of locations with a distinguished initial location such as $\ell_0$, (b) a set of clocks such as $c_0$ (which are initially set to 0), (c) an assignment of a set of atomic propositions (APs) such as $\{done\}$ to each location (for subsequent analysis of e.g. reachability properties), (d) an assignment of constraints on its clocks to each location as invariants such as $c_0 \leq 3$, and (e) a set of probabilistic timed edges each consisting of (e1) a single source location, (e2) at least one target location, (e3) a clock constraint such as $c_0 \geq 2$ specifying as a guard when the edge is enabled based on the current values of the clocks, (e4) for each target location a probability such as 0.5 that this target is reached (the sum of all the probabilities for the target locations of the edge must add up to 1 as a probability distribution is required), and (e5) for each target location a set of clocks such as $\{c_0\}$ to be reset to 0 when that target location is reached.

States of a PTA are given by pairs $(\ell, v)$ where $\ell$ is a location and $v$ is the variable valuation mapping each clock of the PTA to a real number. Nondeterminism arises in PTA since a step for advancing time as well as multiple steps applying rules may be enabled in a single state. The logic PTCTL [17] then allows to specify properties such as "what is the worst-case probability that the PTA reaches a location labeled with the AP *done* within 5 time units", which can be analyzed by the PRISM model checker [16]. For the example PTA from Figure 2d, the given condition is satisfied with probability 0.75 since the nondeterminism of the PTA would be resolved (by a so-called adversary) such that the PTA first takes a step to $\ell_1$ without letting time pass and then performs the probabilistic step (up to two times after waiting for not longer than 2 time units) until it reaches the location $\ell_2$ labeled with the AP *done* (the probabilistic step cannot be taken a third time due to the requirement of at most 5 time units in the quoted property above).

PTGTSs have been introduced in [18, 19] as a probabilistic real-time extension of GTSs. It has been shown that PTGTSs can be translated to PTA and, hence, PTGTSs can be understood as a high-level language for PTA as discussed below in more detail and can be analyzed using PRISM as well.

Similarly to PTA, a PTGTS state is given by a pair $(G, v)$ of a graph and a clock valuation. The initial state is given by a distinguished initial graph and a valuation setting all clocks to 0. In our running example, each attribute of type *clockDrive* of a *Track* node (cf. Figure 2a) represents one clock. Invariants and APs are specified for PTGTSs by means of graph conditions as in Figure 2b and Figure 2e, respectively, for our running example. We use the single invariant $INV_{driving}$ requiring that shuttles in mode *DRIVE* cannot be on a track longer than the value of their *minDur* (minimal duration) attribute plus 1. Moreover, we consider three APs to specify properties that we want to analyze later on. The AP $AP_{unexpectedVelocity}$ is used to detect graphs in which a shuttle does not have an expected velocity of $[2, 3]$ or $[3, 4]$ time units per track where only the lower end of the interval is stored in the graph in the *minDur* attribute. The AP $AP_{collision}$ is used to detect graphs in which two shuttles are on a common track to capture their collision. Finally, the AP $AP_{braked}$ is used to detect graphs in which a shuttle has just executed an emergency brake.

PTGT rules of a PTGTS then correspond to edges of a PTA and contain (a) a left-hand side graph $L$, (b) an attribute constraint on the clock attributes contained in $L$ to capture a guard, (c) a natural number describing a priority where higher numbers denote higher priorities, and (d) a nonempty set of tuples of the form $(\ell : K \hookrightarrow L, r : K \hookrightarrow R, \phi, C, p)$ where $(\ell, r, \phi)$ is an underlying GT rule with application condition $\phi$[1], $C$ is a set of clock attributes contained in $L$ to be reset, and $p$ is a real-valued probability from $[0, 1]$ where the probabilities of all such tuples must add up to 1. See Figure 2f, Figure 2g, and Figure 3a for three PTGT rules *SetSlow*, *ConstructionSiteBrake*, and *Drive* from our running example where the last two PTGT rules have a unique underlying GT rule with probability 1 and where the first PTGT rule has a higher priority as well as two underlying GT rules with probabilities $10^{-6}$ and $1 - 10^{-6}$. For the PTGT rules *ConstructionSiteBrake* and *Drive*, we depict the graphs $L$, $K$, and

[1] The underlying GT rule may not delete or add clock attributes.

$R$ in a single graph (subsequently called *LKR*-graph) where graph elements to be removed and to be added are annotated with $\ominus$ and $\oplus$, respectively. In the PTGT rule *SetSlow*, no graph elements are removed or added (i.e., the graphs $L$ and $R$ of the underlying GT rules coincide). Nevertheless, for this PTGT rule, we depict the two right-hand side morphisms $r_1$ and $r_2$ as they describe PTGT steps with different attribute modifications and probabilities. Also, the PTGT rules *ConstructionSiteBrake* and *Drive* have application conditions, which are depicted left to the $\triangleright$ symbol or above the $\triangledown$ symbol. The attribute preconditions and attribute modifications are given for each PTGT rule in the red box below the *LKR*-graph (or are split into multiple red boxes as for the PTGT rule *SetSlow*). In these attribute preconditions and attribute modifications, unprimed (primed) variables denote the values of attributes before (after) GT rule application. Note that if variables are not changed by the GT rule application, we denote this using the operator *unchanged* (see e.g. Figure 2g where $unchanged(minD_1, tid_1, tid_2)$ denotes that the variables $minD_1$, $tid_1$, and $tid_2$ remain unchanged). Moreover, further information about the PTGT rule (i.e., the guard and the priority) but also further information about the probabilistic choices (i.e., the sets of clocks to be reset and probabilities) are depicted in gray boxes. Lastly, we also allow to annotate a PTGT step in the induced state space with (a) a name chosen for the probabilistic choice such as *success* and *failure* in Figure 2f and (b) the values of the variables contained in the list stepLabel (which may contain variables from $L$ and $R$).

When comparing PTA and PTGTSs, we observe that PTA edges are either enabled for the current valuation or not whereas PTGT rules may be applicable for many matches at the same time (e.g. allowing to apply the *Drive* for one of multiple shuttles). Priorities used in PTGTSs can be encoded in GTSs (including PTGTSs) by adding the left-hand side graphs of rules with higher priorities as negative application conditions to all rules with a lower priority. Similarly, priorities, if integrated into PTA, could be encoded by refining the guards. However, for our running example, we can exchange the underlying track topology without effort, while this would require a fundamental adaptation of the corresponding PTA. Also, as in [19], we observe in section 6 that small PTGTSs result in PTA of considerable size and we therefore conclude that PTGTSs are typically much more concise compared to PTA.

## 4 Decomposition of Large-Scale Topologies

We now present our decomposition-based approach to analyze a PTGTS $\mathcal{S}_0$ modeling a large-scale cyber-physical system along the lines of the informal presentation from the introduction. For our running example, such a PTGTS is given by an initial graph typed over the type graph from Figure 2a that is restricted later on in a suitable way, 13 PTGT rules of which we present three in Figure 2f, Figure 2g, and Figure 3a (further rules are given in [20, Appendix]), the invariant from Figure 2b, and the three APs from Figure 2e.

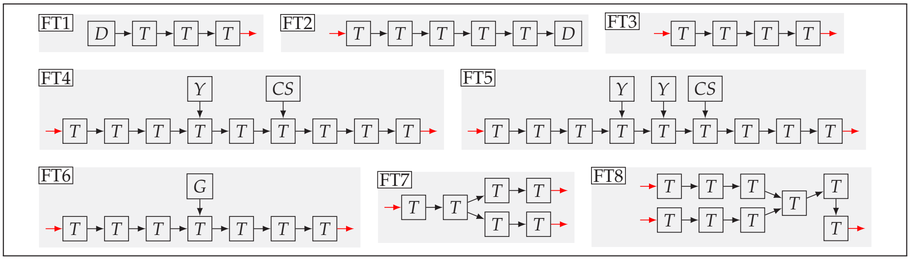

(a) FTs for our running example where the red arrows indicate points for topology (de)composition.

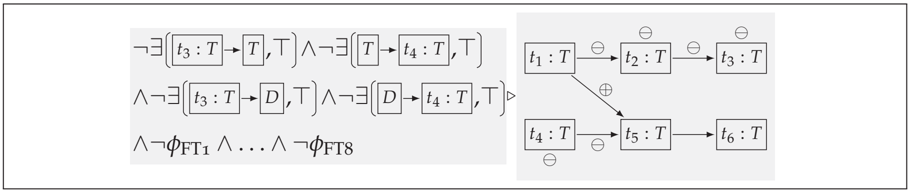

(b) Rule *Merge* for binary overlapping of two instances of FTs.

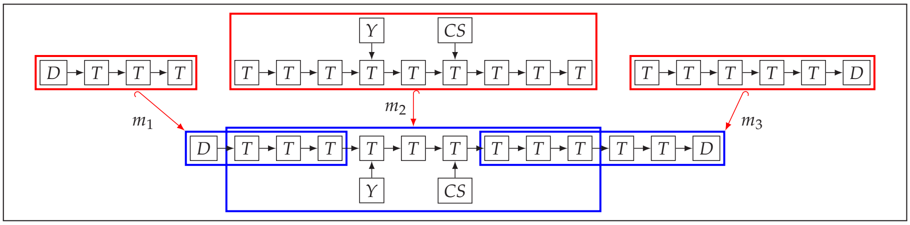

(c) Decomposition $M = \{m_1, m_2, m_3\}$ of an LST w.r.t. FT1–FT8.

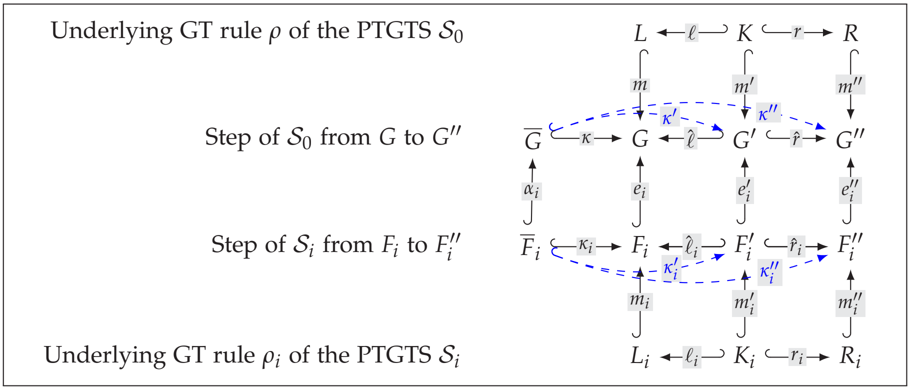

(d) Correspondence of the graph transformation based steps between the large-scale system $\mathcal{S}_0$ and one of its fragment systems $\mathcal{S}_i$, which are preserving the respective static structure given by $\overline{G}$ and $\overline{F}_i$.

Fig. 4: FTs for our running example, rule *Merge*, example for topology composition, and correspondence between steps in the large-scale system and a fragment system.

As a first step, we identify a substructure of the initial graph of $\mathcal{S}_0$ that is static in the sense that this substructure is preserved and also never extended throughout all PTGT steps of $\mathcal{S}_0$. For large-scale cyber-physical systems such as our running example, the existence of such a static substructure may be justified by a logical or spatial distribution. The embedding of a static substructure $\overline{G}$ in a given graph $G$ is then captured by a monomorphism $\kappa : \overline{G} \hookrightarrow G$ describing how $\overline{G}$ is embedded into $G$. As a special case, such an embedding $\kappa$ can be derived for arbitrary graphs $G$ by a monomorphism $\kappa_{TG} : \overline{TG} \hookrightarrow TG$ describing how the given type graph $TG$ is restricted to a smaller type graph $\overline{TG}$. That is, $\overline{G}$ then contains only those elements from $G$ that are typed over the smaller type graph $\overline{TG}$. For our running example, we restrict the type graph $TG$ from Figure 2a to such a smaller type graph $\overline{TG}$ by removing the *Shuttle* node with its attributes, the *at* edge connected to the *Shuttle* node, and the *active* attributes from the *TLYellow* and *TLGreen* nodes. The graphs $\overline{G}$ obtained from graphs $G$ of $\mathcal{S}_0$ using this restriction are then called *large-scale topologies (LSTs)* and contain for our running example a track topology with depots, traffic lights, and construction sites. Note that the fact that such an underlying LST is indeed preserved and never extended by arbitrary rule applications can be verified (at least for our running example) by inspecting each rule individually using the technique of 1-induction [9, 26].

As a second step, we now introduce the notion of a decomposition of the LST into a small set of (constrained) *fragment topologies (FTs)*. Such (constrained) FTs are given by (a) a graph that is typed over the type graph used for the LST and (b) a graph condition describing constraints on how the graph of the FT may be embedded into graphs of $\mathcal{S}_0$. Moreover, an *overlapping specification o* is required to describe how the *embeddings* $\alpha_i$ of the graphs of two FTs may overlap in the LST. Such an overlapping specification is given by a set of spans $(o_1 : O \hookrightarrow \overline{T}_1, o_2 : O \hookrightarrow \overline{T}_2)$ where $O$ is the *permitted overlapping graph* that is embedded into the two FTs. A decomposition of an LST (in the following definition, we simply consider the LST contained in the initial graph $G_0$ of $\mathcal{S}_0$) is then given by embeddings of selected FTs into the LST (cf. Figure 1) such that the overlapping specification is satisfied (the constraints of the FTs are checked for $\mathcal{S}_0$ later on). In applications, to reduce the state space explosion problem for the model checking phase later on, it is advantageous to employ a low number of small FTs that are strictly constrained and are allowed to overlap in a manageable number of ways.

**Definition 1 (Decomposition of LST).** *If*

- *$\mathcal{S}_0$ is a PTGTS with initial state $s_0 = (G_0, v_0)$,*
- *$\kappa : \overline{G}_0 \hookrightarrow G_0$ is a monomorphism identifying the LST of $\mathcal{S}_0$ contained in $G_0$,*
- *$\mathcal{F}$ is a set of (constrained) FTs of the form $(\overline{F}_i, \phi_i)$,*
- *$o((\overline{F}_1, \phi_1), (\overline{F}_2, \phi_2)) \subseteq \{(o_1, o_2) \mid o_1 : O \hookrightarrow \overline{F}_1, o_2 : O \hookrightarrow \overline{F}_2\}$ is an overlapping specification, which describes how two FTs from $\mathcal{F}$ may overlap,*
- *$M$ is a list of tuples of the form $(\overline{F}, \phi, \alpha)$ where $(\overline{F}, \phi) \in \mathcal{F}$ and $\alpha : \overline{F} \hookrightarrow \overline{G}_0$,*
- *the monomorphisms in $M$ respect the overlapping specification $o$, i.e., (see [20] for a visualization) for all $(\overline{F}_1, \phi_1, \alpha_1 : \overline{F}_1 \hookrightarrow \overline{G}_0), (\overline{F}_2, \phi_2, \alpha_2 : \overline{F}_2 \hookrightarrow \overline{G}_0) \in M$ there*

*is some pair* $(o_1 : O \hookrightarrow \overline{F}_1, o_2 : O \hookrightarrow \overline{F}_2) \in o((\overline{F}_1, \phi_1), (\overline{F}_2, \phi_2))$ *such that for the pushout* $(g_1 : \overline{F}_1 \hookrightarrow P, g_2 : \overline{F}_2 \hookrightarrow P)$ *of* $(o_1, o_2)$ *(i.e., the overlapping of* $\overline{F}_1$ *and* $\overline{F}_2$ *w.r.t.* $(o_1, o_2)$*) there is some* $h : P \hookrightarrow G'_0$ *such that* $\alpha_1 = h \circ g_1$ *and* $\alpha_2 = h \circ g_2$.

*then M is a decomposition of the LST of* $\mathcal{S}_0$ *w.r.t.* $\kappa$, $\mathcal{F}$, *and* $o$. ♦

To provide a better intuition for this definition, we now present the decomposition of the LST considered for our running example.

*Example 1 (Decomposition for Running Example).* Let $\mathcal{F}$ contain the constrained FTs (FT$i, \phi_i$) for $1 \leq i \leq 8$ where each FT$i$ is given in Figure 4a (here we use an abbreviated notation where $D$, $T$, $Y$, $G$, and $CS$ are the obvious abbreviations for the node types of the type graph) and where $\phi_i$ states in each case that shuttles must have a velocity of $[2, 3]$ or $[3, 4]$ time units per track.[2]

Let $o((\overline{F}_1, \phi_1), (\overline{F}_2, \phi_2))$ be the overlapping specification stating that overlappings $(o_1 : O \hookrightarrow \overline{F}_1, o_2 : O \hookrightarrow \overline{F}_2)$ of two FTs are always (for any of the $8 \times 8$ combinations) of the form $O = T_1 \to T_2 \to T_3$ where $T_1$ and $T_3$ are mapped to a *Track* node in $\overline{F}_1$ and $\overline{F}_2$ with an entering and an exiting red arrow by $o_1$ and $o_2$, respectively.

An example of a decomposition of an LST employing the previously mentioned FTs and overlapping specification is given in Figure 4c where three FTs are embedded into an LST. To be appropriate later on, the decomposition must ensure that all tracks of the LST are covered by embedding morphisms to which *Shuttle* nodes may be connected (e.g. due to *Shuttle* nodes in the initial graph of $\mathcal{S}_0$ or due to connected *Depot* nodes from which *Shuttle* nodes may enter the LST). In fact, the eight chosen FTs limit the reasoning for our running example to LSTs that can be decomposed using these FTs. ♢

In general, we consider the two use cases: (a) a given PTGTS with underlying LST is to be analyzed and (b) LSTs are to be constructed based on the selected and analyzed FTs. Both use cases are supported but require a different handling. For the use case (a) a parsing of the LST w.r.t. the given FTs and overlapping specification must be performed to obtain a decomposition of the LST. Efficient parsing algorithms have been devised for the special case of hyperedge replacement (HR) grammars (which require that nodes are not deleted) in [8, 6, 7]. A suitable graph transformation based grammar for our running example with 25 rules is given in [20, Appendix]. For the use case (b) in which we need to construct some LST, we may employ node deleting rules. For our running example, consider the rule *Merge* from Figure 4b that can be used to iteratively overlap two FTs starting with a disjoint union of copies of FTs. The rule *Merge* overlaps two instances of three successive *Track* nodes following the overlapping specification where the application condition ensures that the rule is applied at entry and exit points also excluding the possibility that the six matched *Track* nodes belong to an instance of FT$i$ using $\neg\phi_{\mathrm{FT}i}$.

[2] For each FT from Figure 4a, this constraint can be formalized as a graph condition.

## 5 Overapproximation of Behavior

The decompositions of LSTs introduced in the previous section are now used as a foundation to establish a behavioral relationship between a given PTGTS $\mathcal{S}_0$ and $n$ PTGTs $\mathcal{S}_i$ that operate on the instances of FTs that are embedded into the LST of $\mathcal{S}_0$ according to the given LST decomposition.

For this purpose, we extend the structural embeddings given by the $\alpha$ monomorphisms from FTs to the LST in Definition 1 to embeddings of the entire graph (including the static but also the dynamic parts) of a state of some $\mathcal{S}_i$ called *fragment topology state (FTS)* into the entire graph of a state of $\mathcal{S}_0$ called *large-scale state (LSS)*. Consider the left middle square in Figure 4d where the embedding $\alpha_i$ together with the FT and LST embeddings $\kappa_i$ and $\kappa$ is complemented with an embedding $e_i$ of the FTS $F_i$ into the LSS $G$. Note that $e_i$ must be an extension of $\alpha_i$ in the sense that the square commutes (i.e., $\kappa \circ \alpha_i = e_i \circ \kappa_i$ is required). Also, $e_i \circ \kappa_i$ must satisfy the constraint $\phi_i$ of the FT used for $\mathcal{S}_i$.

To simplify our presentation, we assume that the PTGTS $\mathcal{S}_0$ (as in our running example) only employs APs of the form $\exists(f : \emptyset \hookrightarrow P, \top)$, invariants of the form $\neg\exists(f : \emptyset \hookrightarrow P, \top)$, and application conditions in PTGT rules that are conjunctions of graph conditions of the form $\neg\exists(f : \emptyset \hookrightarrow P, \top)$ for some graph $P$. This restriction simplifies the identification of parts of FTSs and LSSs that are considered for an evaluation of such graph conditions.

As a next step, we present a decomposition relation, which establishes a relationship between $\mathcal{S}_0$ and the PTGTSs $\mathcal{S}_i$ in terms of embedding monomorphisms $\kappa$, $\alpha_i$, $e_i$, and $\kappa_i$ for all reachable states of $\mathcal{S}_0$. Moreover, the decomposition relation requires that (a) the timed and discrete steps of $\mathcal{S}_0$ can be mimicked by each affected $\mathcal{S}_i$ and (b) that discrete steps performed by some PTGTS $\mathcal{S}_i$ in isolation on a part of the LST where the FT $\overline{F}_i$ does not overlap with the FT $\overline{F}_j$ of another PTGTS $\mathcal{S}_j$ with $i \neq j$ can be mimicked by $\mathcal{S}_0$. That is, the decomposition relation is a simulation for the steps performed by $\mathcal{S}_0$ and a bisimulation on those steps that are performed in isolation by a single PTGTS $\mathcal{S}_i$. Also, to allow to derive results for $\mathcal{S}_0$ from a model checking based analysis of the PTGTSs $\mathcal{S}_i$, we require a set of APs $\mathcal{A}$ that is part of the APs of $\mathcal{S}_0$ and of each $\mathcal{S}_i$. Based on this set $\mathcal{A}$, the decomposition relation also requires that only those FTSs and LSSs are related that satisfy the same sets of APs in $\mathcal{A}$. For our running example, this set will contain all three APs of $\mathcal{S}_0$ (see Figure 2e). Finally, we require that the initial states of $\mathcal{S}_0$ and the $n$ PTGTSs $\mathcal{S}_i$ are covered by the decomposition relation.

**Definition 2 (Decomposition Relation).** *Given*

- (PTGTS FOR LARGE-SCALE SYSTEM) $\mathcal{S}_0$ *is a PTGTS with initial LSS* $s_0 = (G_0, v_0)$ *where the LST is identified via* $\kappa_0 : \overline{G}_0 \hookrightarrow G_0$ *(and preserved by all steps of the PTGTS),*
- (PTGTSs FOR FTs) *for each* $1 \leq i \leq n$*:* $\mathcal{S}_i$ *is a PTGTS with initial FTS* $s_{0,i} = (F_{0,i}, v_{0,i})$ *where the underlying FT is identified via* $\kappa_i : \overline{F}_{0,i} \hookrightarrow F_{0,i}$ *(and preserved by all steps of the PTGTS),*

- (PRESERVED ATOMIC PROPOSITIONS) $\mathcal{A}$ *is a set of APs contained in each* $\mathcal{S}_i$*, and*
- (DECOMPOSITION OF THE LST) $M$ *is a decomposition of size* $n$ *of the LST of* $\mathcal{S}_0$ *w.r.t.* $\kappa_0$, $\mathcal{F} = \{\overline{F}_{0,i} \mid 1 \leq i \leq n\}$*, and some overlapping specificiation* $o$ *(cf. Definition 1).*

*S is a* decomposition relation *between* $\mathcal{S}_0$ *and* $(\mathcal{S}_1, \ldots, \mathcal{S}_n)$ *containing tuples of the form* $((G, v), \kappa : \overline{G} \hookrightarrow G, w)$ *where* $(G, v)$ *is a state of* $\mathcal{S}_0$*,* $\kappa$ *identifies the LST of G, and w is a tuple of length n of tuples of the form* $(s_i, \overline{F}_i, \phi_i, \alpha_i, \kappa_i, e_i)$ *when the following items are satisfied.*

1. (ELEMENTS OF DECOMPOSITION RELATION) *The relation S explains how the FTS of the PTGTS* $\mathcal{S}_i$ *is embedded into the LSS of* $\mathcal{S}_0$*, i.e., (see Figure 4d) if* $((G, v), \kappa : \overline{G} \hookrightarrow G, w) \in S$ *and* $((F_i, v_i), \overline{F}_i, \phi_i, \alpha_i, \kappa_i, e_i)$ *is the ith element of w, then* $s_i = (F_i, v_i)$ *is a state of* $\mathcal{S}_i$*,* $(\overline{F}_i, \phi_i, \alpha_i)$ *is the ith element of M,* $\kappa_i : \overline{F}_i \hookrightarrow G'$ *(embedding of FT into LST),* $e_i : F_i \hookrightarrow G$ *(embedding of FTS into LSS),* $e_i \circ \kappa_i$ *satisfies* $\phi_i$*, and* $\kappa \circ \alpha_i = e_i \circ \kappa_i$ *(embedding* $e_i$ *is an extension of embedding* $\kappa_i$*),*
2. (CONSISTENT VALUATIONS) *The clock valuations of each FTS agree with the LSS, i.e., if* $((G, v), \kappa : \overline{G} \hookrightarrow G, w) \in S$*,* $((F_i, v_i), \overline{F}_i, \phi_i, \alpha_i, \kappa_i, e_i) \in w$*, and* $\kappa_i(c_i) = c$*, then* $v_i(c_i) = v(c)$*.*
3. (INITIAL STATES RELATED) *The initial LSS of* $\mathcal{S}_0$ *is related, i.e.,* $(s_0, \kappa_0, w) \in S$ *for some w where the ith element* $(s_i, \overline{F}_i, \phi_i, \alpha_i, \kappa_i, e_i)$ *of w satisfies* $s_i = s_{0,i}$*.*
4. (ATOMIC PROPOSITIONS) *The labeling with APs is in agreement w.r.t.* $\mathcal{A}$*, i.e., if* $((G, v), \kappa : \overline{G} \hookrightarrow G, w) \in S$*,* $ap = \exists(f : \emptyset \hookrightarrow P, \top) \in \mathcal{A}$*, the monomorphism* $k : P \hookrightarrow G$ *shows that ap is satisfied by G, then there is some* $1 \leq i \leq n$ *such that* $((F_i, v_i), \overline{F}_i, \phi_i, \alpha_i, \kappa_i, e_i)$ *is the ith element of w, and there is some* $k_i : P \hookrightarrow F_i$ *showing that ap is satisfied by* $F_i$ *and* $k = e_i \circ k_i$*.*
5. (BISIMULATION OF TIMED STEPS) *If* $((G, v), \kappa : \overline{G} \hookrightarrow G, w) \in S$ *and* $\mathcal{S}_0$ *has a timed step (not involving a PTGTS rule) from* $(G, v)$ *to* $(G, v + \delta)$ *then there is some* $((G, v + \delta), w') \in S$ *where* $w'$ *is obtained pointwise from w by applying the corresponding timed step to each* $((F_i, v_i), \overline{F}_i, \phi_i, \alpha_i, \kappa_i, e_i) \in w$ *resulting in* $((F_i, v_i + \delta), \overline{F}_i, \phi_i, \alpha_i, \kappa_i, e_i)$ *and vice versa for a common timed step of each* $\mathcal{S}_i$ *of duration* $\delta$*.*
6. (SIMULATION OF STRUCTURAL STEPS OF $\mathcal{S}_0$ BY $\mathcal{S}_i$) *if*
   - $((G, v), \kappa : \overline{G} \hookrightarrow G, w) \in S$ *and*
   - $\mathcal{S}_0$ *performs the structural step from* $(G, v)$ *to* $(G'', v'')$ *using an underlying GT rule* $\rho = (\ell : K \hookrightarrow L, r : K \hookrightarrow R, \phi_{ac})$ *given in Figure 4d where, since the step of* $\mathcal{S}_0$ *preserves the LST, there are unique* $\kappa' : \overline{G} \hookrightarrow G'$ *and* $\kappa'' : \overline{G} \hookrightarrow G''$ *such that* $\hat{\ell} \circ \kappa' = \kappa$ *and* $\kappa'' = \hat{r} \circ \kappa'$*, then*
   - $((G'', v''), \kappa'' : \overline{G} \hookrightarrow G, w'') \in S$ *for some* $w''$ *that is obtained pointwise from w by adapting each tuple* $((F_i, v_i), \overline{F}_i, \phi_i, \alpha_i, \kappa_i, e_i) \in w$ *into a resulting tuple* $((F''_i, v''_i), \overline{F}_i, \phi_i, \alpha_i, \kappa''_i, e''_i)$ *as follows. If* $m(L) \cap e_i(F_i) = \emptyset$*, then all components of the tuple remain unchanged. Otherwise, the PTGTS* $\mathcal{S}_i$ *must simulate the step and the tuple needs the updating described in the following steps.*
     - *There must be a step of* $\mathcal{S}_i$ *as given in Figure 4d from* $F_i$ *to* $F''_i$ *for some underlying rule* $\rho_i = (\ell_i : K_i \hookrightarrow L_i, r_i : K_i \hookrightarrow R_i, \phi_{ac,i})$ *with the same probability and priority as* $\rho$*.*

- *Since the step of $\mathcal{S}_i$ preserves the FT, there are unique $\kappa'_i : \overline{F}_i \hookrightarrow F'_i$ and the required $\kappa''_i : \overline{F}_i \hookrightarrow F''_i$ such that $\hat{\ell}_i \circ \kappa'_i = \kappa_i$ and $\kappa''_i = \hat{r}_i \circ \kappa'_i$.*
- *The step of $\mathcal{S}_i$ must allow for $e'_i : F'_i \hookrightarrow G'$ and $e''_i : F''_i \hookrightarrow G''$ such that $\hat{\ell} \circ e'_i = e_i \circ \hat{\ell}_i$ and $\hat{r} \circ e'_i = e''_i \circ \hat{r}_i$.*

7. (SIMULATION OF STRUCTURAL STEPS OF $\mathcal{S}_i$ ON ITS CORE BY $\mathcal{S}_0$) *if*
   - $((G, v), \kappa : \overline{G} \hookrightarrow G, w) \in S$,
   - $((F_i, v_i), \overline{F}_i, \phi_i, \alpha_i, \kappa_i, e_i) \in w$,
   - *$\mathcal{S}_i$ performs the structural step from $(F_i, v_i)$ to $(F''_i, v''_i)$ using an underlying GT rule $\rho_i = (\ell_i : K_i \hookrightarrow L_i, r_i : K_i \hookrightarrow R_i, \phi_{ac,i})$ given in Figure 4d where, since the step of $\mathcal{S}_i$ preserves the FT, there are unique $\kappa'_i : \overline{F}_i \hookrightarrow F'_i$ and $\kappa''_i : \overline{F}_i \hookrightarrow F''_i$ such that $\hat{\ell}_i \circ \kappa'_i = \kappa_i$ and $\kappa''_i = \hat{r}_i \circ \kappa'_i$,*
   - *$e_i(m_i(L_i))$ does not overlap with any $e_j(F_j)$ for $i \neq j$, then*
   - *there is some $((G'', v''), \kappa'' : \overline{G} \hookrightarrow G, w'') \in S$ for some $G''$, $v''$, $\kappa''$, and $w''$ as follows.*
     - *There must be a step of $\mathcal{S}_0$ as given in Figure 4d from $G$ to $G''$ for some underlying rule $\rho = (\ell : K \hookrightarrow L, r : K \hookrightarrow R, \phi_{ac})$ with the same probability and priority as $\rho_i$.*
     - *Since the step of $\mathcal{S}_0$ preserves the LST, there are unique $\kappa' : \overline{G} \hookrightarrow G'$ and the required $\kappa'' : \overline{G} \hookrightarrow G''$ such that $\hat{\ell} \circ \kappa' = \kappa$ and $\kappa'' = \hat{r}_i \circ \kappa'$.*
     - *The step of $\mathcal{S}_0$ must allow for $e'_i : F'_i \hookrightarrow G'$ and $e''_i : F''_i \hookrightarrow G''$ such that $\hat{\ell} \circ e'_i = e_i \circ \hat{\ell}_i$ and $\hat{r} \circ e'_i = e''_i \circ \hat{r}_i$.*
     - *Finally, $w''$ is obtained from $w$ by only adapting the above chosen tuple $((F_i, v_i), \overline{F}_i, \phi_i, \alpha_i, \kappa_i, e_i)$ into the tuple $((F''_i, v''_i), \overline{F}_i, \phi_i, \alpha_i, \kappa''_i, e''_i)$.* ♦

We now state that decomposition relations allow for the simulation of each path of the PTGTS $\mathcal{S}_0$ by the PTGTSs $\mathcal{S}_i$.

**Lemma 1 (Existence of Simulating Paths).** *If $S$ is a decomposition relation between $\mathcal{S}_0$ and $(\mathcal{S}_1, \ldots, \mathcal{S}_n)$, and $\pi$ is a path of length $m$ in $\mathcal{S}_0$ from the initial state to a state $s_m$, then, for each $1 \leq i \leq n$, there is a path $\pi_i$ of $\mathcal{S}_i$ (of length $k_i \leq m$) ending in a state $s_{i,k_i}$ such that $(s_m, \kappa, w) \in S$ for some $\kappa$ and $w$ where the ith element of $w$ is of the form $(s_{i,k_i}, \overline{F}_i, \phi_i, \alpha_i, \kappa_i, e_i)$. Moreover, the probability of each such path $\pi_i$ is at least as high as the probability of the path $\pi$. See [20] for the proof.*

We now state that a PTGTS satisfies a safety property given by an AP, when safety w.r.t. this AP can be established for each $\mathcal{S}_i$.

**Theorem 1 (Safety Verification).** *If $S$ is a decomposition relation between $\mathcal{S}_0$ and $(\mathcal{S}_1, \ldots, \mathcal{S}_n)$ w.r.t $\mathcal{A}$ and $ap \in \mathcal{A}$, then $\mathcal{S}_0$ is safe w.r.t. the occurrence of an ap-labeled graph when (for each $1 \leq i \leq n$) $\mathcal{S}_i$ is safe w.r.t. the occurrence of an ap-labeled graph. Moreover, the probability of an occurrence of an ap-labeled graph from some state $s$ in $\mathcal{S}_0$ is smaller than the probability of an occurrence of an ap-labeled graph from some S-related state $s_i$ in $\mathcal{S}_i$. See [20] for the proof.*

We now apply the proposed methodology of establishing a behavioral relationship between the PTGTS $\mathcal{S}_0$ and the PTGTSs $\mathcal{S}_i$ to our running example. For this purpose, we now describe how the FTS of each $\mathcal{S}_i$ is embedded into the LSS of $\mathcal{S}_0$ and, based on this embedding, how the $\mathcal{S}_i$ is derived from $\mathcal{S}_0$.

*Example 2 (Construction of Embeddings and Simulating PTGTSs).* Firstly, the embeddings $e_i$ of FTSs into the LSS are obtained as extensions of the structural embeddings $\kappa_i$ by also matching (a) all *Shuttle* nodes (with their attributes) that are connected to *Track* nodes contained in the FT via *next* edges and (b) all *active* attributes of *TLYellow* and *TLGreen* nodes contained in the FT. This extension also naturally applies to the initial state of $\mathcal{S}_0$. Clearly, two embeddings $e_i$ and $e_j$ (for $i \neq j$) only overlap in elements of their FTs but not in the additionally matched dynamic elements.

Secondly, we adapt the given PTGTS $\mathcal{S}_0$ to obtain for each of the eight FTs one PTGTS $\mathcal{S}_i$ by (a) changing the initial graph to the source of $e_i$ capturing the FT as well as the additional dynamic elements of the initial state of $\mathcal{S}_0$ connected to it, (b) adding eight rules for overapproximating the behavior of $\mathcal{S}_0$ on the tracks that may overlap with tracks of other FTs. For the latter point, we observe that all but three of the rules of $\mathcal{S}_0$ (including *SetSlow* and *ConstructionSiteBrake* from Figure 2) are never applicable on the parts of FTs that may overlap with other FTs (i.e., borders of FTs). The remaining three rules are *Drive* from Figure 3a as well as two similar rules for stopping the shuttle that we do not consider in detail here. Three of the four derived rules for rule *Drive* are given in Figure 3.

The additional rule *DriveEnterFast* is used to simulate *Drive* steps where a shuttle in $\mathcal{S}_0$ drives from a track not covered by $\mathcal{S}_i$ to a track covered by $\mathcal{S}_i$. The rule *DriveEnterFast* is essentially constructed by omitting the source track $T_1$ from the rule *Drive*, by adding the shuttle with one of the two expected velocities (the other velocity results in the omitted rule *DriveEnterSlow*)[3], and by omitting application conditions that may not be satisfied due to the overlapping specification and the structure of FTs.

Similarly, the additional rules *DriveExit1* and *DriveExit2* are constructed from rule *Drive* to allow for the simulation of the two steps in which a shuttle in $\mathcal{S}_0$ drives using rule *Drive* on two tracks covered by $\mathcal{S}_i$ to a track not covered by $\mathcal{S}_i$. These two rules are then constructed similarly, by omitting the tracks $T_3$ (for *DriveExit1*) and $T_3$ and $T_4$ (for *DriveExit2*) from rule *Drive* as these are not covered by the $\mathcal{S}_i$, by removing the shuttle with its attributes in rule *DriveExit2*, by omitting application conditions that may not be satisfied due to the overlapping specification and the structure of FTs, and by omitting application conditions that refer to the removed tracks.

Note that these additional rules overapproximate the behavior that is possible in $\mathcal{S}_0$ as they may be used when analyzing $\mathcal{S}_i$ also when no corresponding shuttle in $\mathcal{S}_0$ is able to enter the FT or when rule *Drive* would be disabled due to the omitted application conditions for the case of rules *DriveExit1* and *DriveExit2*. ♢

For our running example, we now describe the construction of a suitable decomposition relation relying on the LST decomposition introduced before.

[3] Here, we rely on the constraints on the eight FTs (cf. Example 1) requiring that the AP $AP_{unexpectedVelocity}$ is never labeled in the large-scale system $\mathcal{S}_0$.

**Lemma 2 (Existence of Decomposition Relation for Running Example).** *For the PTGTS* $\mathcal{S}_0$ *of our running example with an arbitrary initial LST such that M is a decomposition of that LST w.r.t. some monomorphism* $\kappa$*, the set of eight FTs, and the overlapping specification o from Example 1 there is a decomposition relation S between* $\mathcal{S}_0$ *and the n PTGTSs* $\mathcal{S}_i$ *from Example 2. See [20] for the proof.*

Based on this decomposition relation and Theorem 1, we can obtain the desired overapproximation result for $\mathcal{S}_0$ for the qualitative safety w.r.t. collisions and the quantitative unlikeliness of emergency brakes.

**Corollary 1 (Qualitative and Quantitative Safety for Running Example).** $\mathcal{S}_0$ *exhibits no collisions when this is the case for each* $\mathcal{S}_i$*. Moreover, emergency brakes are performed in* $\mathcal{S}_0$ *with a probability not higher than the probability of such an occurrence in any* $\mathcal{S}_i$*.*

Note that we only need to analyze one PTGTS for each of the eight permitted FTs w.r.t. the occurrence of collisions and the probability of emergency brakes.

## 6 Evaluation

To analyze the eight PTGTSs constructed for our running example in section 5 (see Table 1 for the results), we have employed the methodology from [19] generating the state spaces for these PTGTSs without timed steps and then generated the corresponding PTA from these state spaces. We then restricted these PTA to timed automata (TA) essentially removing the information on probabilities, applied UPPAAL [15] to determine the edges of the TA that can never be applied due to unsatisfiable guards, and removed the corresponding edges from the previously generated PTA. The entire analysis using our prototypical implementation required less than three days on a machine using up to 250 GB memory where the state space generation required most of the time. However, there is a vast potential for optimizations regarding memory consumption (by only storing subsequently relevant information on states and steps) and runtime (by facilitating concurrency during state space generation).

Firstly, using UPPAAL, we have verified that each of the eight TA (hence, also the eight PTA) have no reachable deadlock (where also timed steps are disabled). Hence, we obtain that the PTGTS $\mathcal{S}_0$ also does not contain this particular modeling error since, using the decomposition relation, we also obtain that every deadlock reachable in $\mathcal{S}_0$ can be reached analogously in each $\mathcal{S}_i$.

Secondly, we have observed that the obtained PTA do not label any location with $AP_{unexpectedVelocity}$ or $AP_{collision}$. For $AP_{unexpectedVelocity}$ this means that the additional rules such as *DriveEnterFast* and *DriveEnterSlow* for overapproximating the steps of entering shuttles entirely cover all possible velocities of shuttles. For $AP_{collision}$ this means that Corollary 1 implies that the PTGTS $\mathcal{S}_0$ with an LST constructed in the described way from the eight FTs is safe w.r.t. the occurrence of collisions.

Thirdly, to verify that yellow traffic lights suitably slow down the shuttles before construction sites, we have identified locations $\ell_i$ in the resulting PTA

Table 1: Results of our evaluation for the running example

| fragment topology | states | steps | collisions | max. probability for violating the velocity limit at a construction site |
|---|---|---|---|---|
| FT1 | 9 | 18 | 0 | 0 |
| FT2 | 335 | 693 | 0 | 0 |
| FT3 | 216 | 503 | 0 | 0 |
| FT4 | 109 379 | 312 915 | 0 | $1 \times 10^{-6}$ |
| FT5 | 106 122 | 284 102 | 0 | $1 \times 10^{-12}$ |
| FT6 | 12 473 | 31 812 | 0 | 0 |
| FT7 | 4048 | 16 314 | 0 | 0 |
| FT8 | 121 953 | 452 340 | 0 | 0 |

that are labeled with $AP_{braked}$ (occurring only in FT4 and FT5). In each case, we were able to track using a custom analysis algorithm (since the PRISM model checker was too slow for the large PTA at hand) the shuttle backwards over all possible paths leading to such a location $\ell_i$ up to the step where the shuttle entered the FT. We then determined the maximal probability of any such path obtaining a worst-case emergency brake probability of $10^{-6}$ and $10^{-12}$ for any entering shuttle in FT4 and FT5, respectively. On the one hand, FT5 is thereby verified to be quantitatively more desirable compared to FT4. On the other hand, Corollary 1 implies that installations of yellow traffic lights as in FT4 and FT5 suitably decrease the likelihood of emergency brakes also for $\mathcal{S}_0$. However, the probabilities that some shuttle executes an emergency brake in a given time span in FT4/FT5 (obtained by combining the maximal throughput of shuttles for FT4/FT5 with the worst-case probability obtained for FT4/FT5) can be expected to be too coarse upper bounds when the maximal throughput is not to be expected for the real system.

## 7 Conclusion and Future Work

We presented an analysis approach for large-scale systems modeled as PTGTSs for which model checking is not feasible. In this approach, we rely on a decomposition of an underlying static large-scale topology into fragment topologies of manageable size. Model checking is then applied for each fragment topology and an adaptation of the PTGTS to such a fragment topology. We thereby determine (a) overapproximations of reachability properties important for qualitative safety properties and (b) upper bounds for probabilistic reachability properties important for quantitative safety properties.

As future work, we intend to extend our analysis to fairness properties and conditions of the metric temporal graph logic (MTGL) [29]. Also, to cover further aspects of the RailCab project [23], we will develop more general decomposition schemes where dynamic components (such as connected shuttles driving in convoys) may be covered by multiple fragment topologies. Lastly, to further evaluate applicability of our approach, we intend to apply it to other case studies as e.g. the one discussed in [1].
